# Ranitidine degradation in layered double hydroxide activated peroxymonosulfate system: impact of transition metal composition and reaction mechanisms

**DOI:** 10.1007/s11356-024-34331-5

**Published:** 2024-07-15

**Authors:** Afia Sharmin, Muhammad Bilal Asif, Guomin Zhang, Muhammed A. Bhuiyan, Biplob Pramanik

**Affiliations:** 1 School of Engineering, RMIT University, Melbourne, VIC 3001 Australia; 2 Advanced Membranes and Porous Materials Center (AMPMC), Physical Sciences and Engineering (PSE), King Abdullah University of Science and Technology (KAUST), Thuwal, 23955 Saudi Arabia

**Keywords:** Layered double hydroxide, Water treatment, Peroxymonosulfate, Ranitidine, Heterogenous catalysis, Reaction pathways

## Abstract

Ranitidine, a competitive inhibitor of histamine H_2_ receptors, has been identified as an emerging micropollutant in water and wastewater, raising concerns about its potential impact on the environment and human health. This study aims to address this issue by developing an effective removal strategy using two types of layered double hydroxide (LDH) catalysts (i.e., CoFeLDH and CoCuLDH). Characterization results show that CoFeLDH catalyst has superior catalytic properties due to its stronger chemical bond compared to CoCuLDH. The degradation experiment shows that 100% degradation of ranitidine could be achieved within 20 min using 25 mg/L of CoFeLDH and 20 mg/L of peroxymonosulfate (PMS). On the other hand, CoCuLDH was less effective, achieving only 70% degradation after 60 min at a similar dosage. The degradation rate constant of CoFeLDH was 10 times higher than the rate constant of CoCuLDH at different pH range. Positive zeta potential of CoFeLDH made it superior over CoCuLDH regarding catalytic oxidation of PMS. The catalytic degradation mechanism shows that sulfate radicals played a more dominant role than hydroxyl radicals in the case of LDH catalysts. Also, CoFeLDH demonstrated a stronger radical pathway than CoCuLDH. XPS analysis of CoFeLDH revealed the cation percentages at different phases and proved the claim of being reusable even after 8 cycles. Overall, the findings suggest that CoFeLDH/PMS system proves to be a suitable choice for attaining high degradation efficiency and good stability in the remediation of ranitidine in wastewater.

**Supplementary Information:**

The online version contains supplementary material available at 10.1007/s11356-024-34331-5.

## Introduction

The presence of micropollutants in water and wastewater has emerged as a major public health concern, particularly due to their frequent detection in wastewater treatment plant effluents. Among these pollutants, pharmaceuticals such as ranitidine are significant and inevitable contributors to the damage of the ecosystem. Ranitidine, a medication used to treat peptic ulcers by inhibiting histamine H_2_ receptors, can have adverse impacts on the aquatic ecosystem and human health after prolonged exposure (Sharmin et al. 2024). Its presence in water systems is therefore especially alarming. Studies reveal that only 30% of ranitidine is metabolized in the human body, while the remaining 70% enters the environment through urine (Rivas et al. 2009). This unmetabolized ranitidine can form nitrosodimethylamine (NDMA), which is a substance significantly more carcinogenic than trihalomethanes, posing a threat to the environment. The presence of carcinogenic compounds, even at trace levels, is harmful (Seid et al. 2021). According to the U.S. and European data, ranitidine concentrations range from 1 to 10 ng/L in surface water and from 220 to 550 ng/L in wastewater (Zhang et al. 2017). As mentioned above, ranitidine can react with disinfectants (such as during the chloramination of water) in drinking water and produce NDMA, which cannot be removed through conventional drinking water treatment processes (Seid et al. 2021). As such, it is essential to remove ranitidine in water and wastewater treatment plants.

Several conventional techniques, such as physicochemical processes (Kabir et al. 2024), biological treatment approaches, and membrane separation, have been employed to remove micropollutants from water and wastewater (Zhang et al. 2017). However, these processes have limitations, such as sludge production during chemical processes, and resistance of micropollutants to biodegradation in biological treatment processes (Väänänen et al. 2016). As a result, effective treatment technologies need to be developed for degrading pharmaceutically active micropollutants including ranitidine. Advanced oxidation processes (AOPs) have become popular for micropollutant elimination and involve the degradation of organic micropollutants through the generation of strong radicals, which have been demonstrated to be effective due to their oxidizing ability, applicability, and reaction rate (Cheng et al. 2016; Xu et al. 2020; Zhu et al. 2019). Notable AOPs include photocatalysis, electrocatalysis, Fenton oxidation, and persulfate oxidation. However, hydroxyl radical (OH^•^)–based Fenton reactions have some limitations, such as a narrow pH range and sludge production (Yao et al. 2014). Peroxymonosulfate (PMS)–based oxidation has attracted attention due to its higher redox potential and the extended half-life of sulfate radicals (SO_4_^•−^) (Gul et al. 2021). SO_4_^•−^ is formed by breaking the S–O bond of PMS via a process facilitated through energy input and electron or charge transfer reactions. Moreover, PMS requires activators like transition metal composites, microwaves, heat, and UV to effectively remove contaminants such as ranitidine (Han et al. 2012; Li et al. 2022; Milh et al. 2020). A previous research study has shown that degradation efficiency of acetaminophen was higher with a layered double hydroxide (LDH) catalyst than without one in a PMS system (Zhu et al. 2019). This statement was justified by many other micropollutants and dyes. However, the difficulty in recovering nano catalysts from aqueous media in conventional AOPs, potential contamination from intermediate products, and the creation of secondary pollution necessitates the development of new strategies.

Among different activators, transition metals can disrupt the S–O bond and transform from a low valence state to a high valence state through electronic charge transfer (Li et al. 2022). However, transition metals present challenges, such as metal leaching and difficulties in recovery after use (Ushani et al. 2020). Commonly used transition metals include Co, Fe, Cu, and Mn, and are utilized as catalysts due to their multiple valence state and high electron transfer capability. Co^2+^ has been proved to show the highest reactivity (Deng et al. 2018). Although recent studies have focused on Co, Mn, Fe/PMS systems, transition metal–based homogeneous PMS systems are impractical due to the difficulties associated with their recovery. These limitations can be overcome by using transition metal–based heterogeneous catalysts. In this context, LDHs can serve as stable and highly efficient heterogeneous catalysts in PMS-based AOP systems. LDH possesses a two-dimensional structure similar to hydrotalcite with subsequent formula: [M_1−×_^2+^ M_*x*_^3+^ (OH)_2_]^*x*+^(A^*n*−^)_*x*/*n*_ • mH_2_O, where M^2+^ and M^3+^ are the divalent (e.g., Cu^2+^ and Co^2+^) and trivalent (e.g., Al^3+^ and Fe^3+^) metal ions with a molar ratio of *x*, respectively, while A^*n*−^ denotes the interlayer anions (e.g., NO_3_^−^ and CO_3_^2−^) (Asif et al. 2022). Their layered structure promotes good water permeability, and they can possess divalent and trivalent metal cations at the corners with hydroxides on the arms and intercalated anions within the structure (Asif et al. 2022). Due to their unique structures, LDH-based catalysts have started gaining momentum for PMS activation.

Transition metal–based homogenous catalysts have been replaced by LDH-based heterogenous catalysts in various applications (Ahn et al. 2019). Several studies have reported micropollutant removal by transition metal–based LDH/PMS systems in AOPs with Co as the best performer compared to other transition metals such as Mn, Fe, and Cu (Ahn et al. 2019). Atomically dispersed catalysts (ADCs), ranging from single-atom to dual-atom configurations, serve as a link between heterogeneous and homogeneous catalysts, bridging the gap between the two (Deng et al. 2024). There are several studies conducted to explore the performance of LDH/PMS system. For instance, CoMnLDH has been investigated to degrade acid orange G dyes (Zhao et al. 2018), while CoFeLDH has been used for cadmium ion removal (Moaty et al. 2017) and fluoroquinolone removal in PMS systems (Yang et al. 2021). CoCuLDH achieved approximately 96% of lomefloxacin removal within 30 min (Guo et al. 2020). CuCoFeLDH (0.1 g/L) and PMS (0.5 mM) have been used for nitrobenzene removal, with degradation completed within 6 min, primarily through hydroxyl radicals (Lu et al. 2019). CoFeLaLDH catalyst (0.05 g/L) and PMS (1.0 mM) have been used in tetracycline degradation, achieving 90% removal within 10 min (Li et al. 2020). MgCuFeLDH (0.3 g/L) and 0.5 mM PMS can degrade 93% of acetaminophen within 20 min (Zhu et al. 2019). CoFeLDH supported on nickel foam (NF) as a reusable activator for peroxymonosulfate (PMS) has been synthesized to effectively decontaminate monomethylehydrazine (Qian et al. 2023). Nitrogen self-doped chitosan carbon aerogel (NCCA) combined with CoAlLDH has been produced for highly efficient activation of peroxymonosulfate (PMS) to degrade sulfamethoxazole (97.5%) within just 5 min (Fu et al. 2023). Thus, various transition metal–based LDH catalysts with specific PMS dosages have been employed to degrade individual micropollutants from water and wastewater with varying degrees of efficiency (Lu et al. 2019; Moaty et al. 2017; Sharmin et al. 2024; Yang et al. 2021; Zhao et al. 2018). This underscores the need to enhance the degradation efficiency of certain micropollutants beyond the results achieved in previous research, which utilized lower concentrations of LDH and PMS over brief intervals. Distinguishing this study from earlier efforts, the current research aims to determine the effects of minimal LDH and PMS quantities in developing a novel nanocatalyst capable of achieving optimal removal efficiency of ranitidine. This involves testing two distinct transition metal compositions in LDH. To the best of our knowledge, although radical and non-radical pathways in the LDH/PMS system have been extensively explored (Guo et al. 2020; Li et al. 2020; Lu et al. 2019; Yang et al. 2021), the influence of transition metal composition on these pathways has been less examined.

The aim of this study was to develop LDH catalysts with different transition metal combinations to achieve more effective removal efficiency of ranitidine than what has been previously reported in the literature (Asif et al. 2022), while also exploring unexplored catalytic mechanisms associated with different transition metals for similar micropollutant degradation.

In this context, CoFeLDH and CoCuLDH were prepared and characterized using various analytical techniques. The effects of catalyst dosage, PMS dosage, and pH on the effectiveness of both LDH powders were investigated to achieve maximum ranitidine degradation. Catalytic mechanism was also determined to understand the effect of transition metal on ranitidine degradation. Additionally, the LDH powders were tested for ranitidine degradation with the presence of anions and humic acid, and their reusability and metal leaching properties were also evaluated to determine their practical application in wastewater remediation. The novelty of this work is to produce a nanocatalyst to achieve the highest removal efficiency of ranitidine with the trial of two different transition metal compositions in LDH.

## Materials and methods

### Chemical and materials

Potassium peroxymonosulfate (PMS, KHSO_5_·0.5KHSO_4_·0.5K_2_SO_4_), cobalt nitrate hexahydrate (Co(NO_3_)_2_·6H_2_O), copper nitrate trihydrate (Cu (NO_3_)_2_·3H_2_O), sodium carbonate (Na_2_CO_3_), sulfuric acid (H_2_SO_4_), hydrochloric acid (HCl), sodium hydroxide (NaOH), iron (III) nitrate nonahydrate (Fe(NO_3_)_3_.9H_2_O), sodium hydroxide (NaOH), sodium nitrate (NaNO_3_), sodium bicarbonate (NaHCO_3_), sodium chloride (NaCl), Tert-butyl alcohol (TBA), ethanol (CH_3_CH_2_OH), methanol (CH_3_OH), p-benzoquinone (BQ), and L-Histidine were supplied by Chem Supply Australia. All chemicals used in this study were of analytical grade (> 99%).

### Synthesis of LDH powder

The bulk CoFeLDH was prepared using a hydrothermal method (Asif et al. 2022). Two solutions were prepared as LDH precursors. The first solution was the dissolution of Co(NO_3_)_2_.6H_2_O (0.6 M) and Fe(NO_3_)_3_.9H_2_O (0.3 M) in Milli-Q water. The second solution was made by mixing NaOH (1.92 M) and Na_2_CO_3_ (0.80 M) in Milli-Q water. Both solutions were moved to a beaker and stirred for 1 h at solution pH 10. The obtained solution comprising the contents of LDH was transferred to a 200-mL Hydrothermal Synthesis Autoclave Reactor with polytetrafluoroethylene** (**PTFE) Lined Vessel and put in an oven at 120 °C for 20 h. The autoclave was kept at room temperature (20 °C) for 3 h to cool naturally. CoFeLDH precipitates were centrifuged with water and ethanol three times each. Finally, CoFeLDH powder was found by making dry in an oven at 80 °C.

CoCuLDH powder was synthesized following the same procedures used for CoFeLDH, except that the first solution was prepared by dissolving Co(NO_3_)_2_.6H_2_O (0.6 M) and Cu(NO_3_)_2_.3H_2_O (0.3 M) in Milli-Q water.

### Material characterizations

For the identification of mineral phases, X-ray diffraction (XRD) analysis was conducted on powder samples prepared in standard sample holders using a Bruker D4 Endeavour, USA. Morphological analysis was carried out using scanning electron microscopy (SEM) (Quanta 2) with energy-dispersive X-ray spectroscopy (EDS) (Quanta 2). SEM–EDS in high vacuum conditions was performed at a 20 kV accelerating voltage and a 7 nm spot size for at least 20 min, using the FEI Quanta 200 SEM equipped with an Oxford EDS detector. Data were collected with AZtec software, and the Map tool in AZtec was used to create a map showing the element distribution. Transmission electron microscopy (TEM) and scanning transmission electron microscopy (STEM) were measured by JEOL F200 Transmission Electron Microscope. Chemical bond analysis was conducted using a PerkinElmer FT-IR/FIR Spectrometer Frontier. The residual ranitidine concentration was measured by a UV2700 Shimadzu UV–vis spectrophotometer. Elemental identification was completed by acquiring X-ray data.

### Degradation experiments

To verify the catalytic performance, batch experiments were carried out in a 100-mL volumetric flask at room temperature (20 ± 2 °C). A 100 mg/L stock solution of ranitidine was prepared, and the selected initial concentration (5 mg/L) was added with a pipette to a 100-mL flask. Distilled water was poured into the flask up to 100 mL. For optimization, 10 to 50 mg/L of CoFeLDH and 10 to 30 mg/L of PMS were added to the volumetric flask from stock solutions. Similar procedures were followed for CoCuLDH with similar dosages of LDH catalyst and PMS. The mixture was stirred with a magnetic stirrer rotating at 200 rpm. At 2-min intervals, samples (2 mL) were taken from the reaction solution and monitored by a UV–vis spectrophotometer (UV2700, Shimadzu Corporation, Japan) at 314 nm to analyze the residual ranitidine concentration. In addition, to test the effect of pH value on the ranitidine solution, pH was adjusted with 0.1 M NaOH or 5% HCl. TBA, methanol, L-histidine, and P-benzoquinone were added to the solution to understand the role of radicals. Inorganic (i.e., anions) and organic substances (i.e., humic acid) were added to ranitidine containing solution for simulating natural water environment and understand their impact on ranitidine degradation. For the recycling experiment, the used CoFeLDH in the suspension was collected by centrifugation, washed several times with Milli-Q water, and dried overnight at 60 °C in a vacuum.

### Analytical methods

Metal leaching was analyzed through Agilent 7700 s inductively coupled plasma mass spectrometry (ICP-MS). All the experiments were executed three times, with a relative standard deviation of less than 10%. Samples were inspected using the HPLC–MS/MS method (LCMS-1260, Agilent technologies) to detect degradation intermediates. A C18 HPLC capillary column (150 × 4.6 mm, 5 μm, Eclipse Plus C18) was used for separation purpose. Dissolved gases were removed by filtering and sonicating the mobile phase before detection. Then, a C18 column was utilized as the stationary phase with a mobile phase. Mobile phase contained methanol (containing 0.1% formic acid) (A) and water (containing 0.1% formic acid) (B) at a flow rate of 0.2 mL/min. Ranitidine was observed by a mass spectrometer through both positive and negative gradient. The capillary voltage was set at 240 V and desolvation gas flow rate was 650 L/h, respectively, at a temperature of 350 °C. Besides, the cone gas flow rate, collision gas flow, and source temperature were organized at 50 L/h, 0.05 mL/min, and 120 °C respectively.

## Result and discussion

### Characterization of CoFeLDH and CoCuLDH powder

**Fig. 1:**
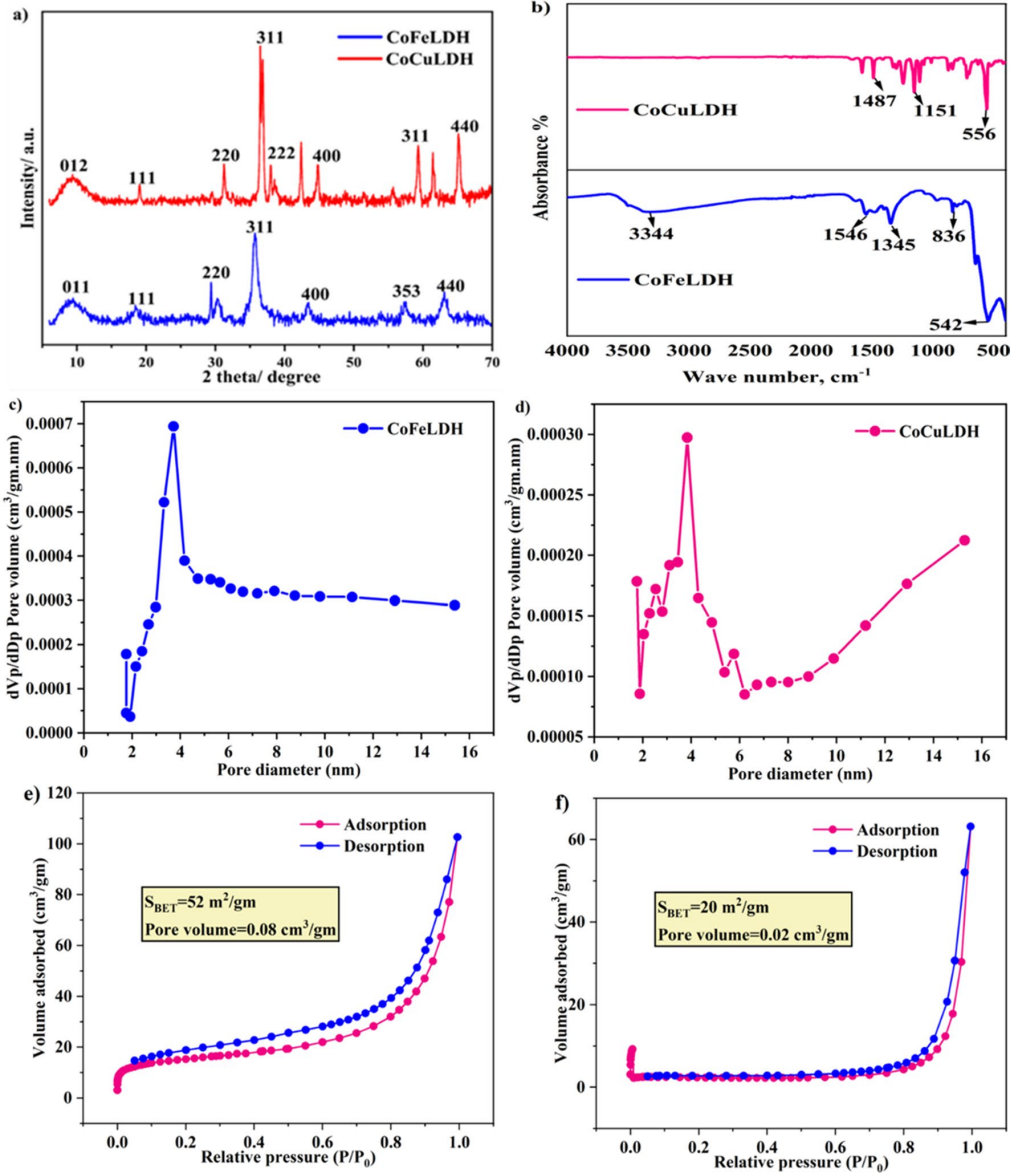
(**a**) XRD of CoFeLDH and CoCuLDH powder, (**b**) FTIR spectra of CoFeLDH and CoCuLDH powder, (**c**) BET analysis of CoFeLDH powder, (**d**) BET analysis of CoCuLDH powder, (**e**) adsorption desorption isotherm of CoFeLDH, and (**f**) adsorption desorption isotherm of CoCuLDH

XRD diffractograms were obtained to assess the crystal structure of CoFeLDH powder. Characteristic peaks for CoFeLDH powder were observed at 18°, 29°, 30°, 36°, and 43° (Fig. 1a), denoting the hexagonal structure from Bragg’s reflections (Mutharasi et al. 2021; Xu et al. 2021). The high intensity of the reflections indicated that the material was highly crystalline. Two small peaks were found between 55 and 65°, which may be due to the interlayer anions. Similar findings were reported for CoFeLDH (Yang et al. 2021). LDH possesses a hexagonal structure with several layers, and the d-spacing of its planes indicates the spacing between adjacent layers. A smaller d-spacing means a narrower layer structure (Zhang et al. 2020). Characteristic peaks for CoCuLDH powder were observed at 10°, 20°, 32°, 37°, 42°, 45°, 59°, 62°, and 65° (Fig. 1a), and these angles indicated 011, 111, 220, 311, 222, 400, 353, and 440 Bragg reflections, respectively. These indicate a hexagonal structure from Bragg’s reflections. Frequent peaks with high intensity demonstrate that it is a material with high crystallinity. Peaks found at 59°, 62°, and 65° indicate the presence of several interlayer anions. Both CoFeLDH and CoCuLDH were confirmed as crystalline materials from XRD analysis.

FTIR spectrum for CoFeLDH is shown in Fig. 1b. Characteristic bands representing chemical bond information were found at 3232 cm⁻^1^, 1634 cm⁻^1^, 1339 cm⁻^1^, 835 cm⁻^1^, and 542 cm⁻^1^. Similar findings were reported for CoFeLDH bands in the ranges of 3200–3400 cm⁻^1^, 1340–1380 cm⁻^1^, and 584 cm⁻^1^ (Yang et al. 2021) (Badakhshan et al. 2019). The former band (i.e. 3232 cm⁻^1^) may be attributed to the vibration of the hydroxyl bond and a few water molecules (Gong et al. 2017; Mutharasi et al. 2021). The middle band (1634 cm⁻^1^ and 1339 cm⁻^1^) may be caused by the vibration of intercalated NO₃⁻ and CO₃^2^⁻ ions (Badakhshan et al. 2019; Hou et al. 2019). The peak in the wavelength range less than 600 indicates the presence of metal oxides (Gong et al. 2017). Characteristic bands for CoCuLDH, representing chemical bond information, were found at 1487 cm⁻^1^, 1151 cm⁻^1^, and 556 cm⁻^1^ (Fig. 1b). The most clear and intense peak at 556 cm⁻^1^ indicates that metal oxides are present in CoCuLDH. Evidence of intercalated anions is also found in CoCuLDH, but the transmittance is much lower than that of CoFeLDH. The peak for the hydroxyl bond and a few water molecules is very low for CoCuLDH. Lower hydroxyl bond content may create difficulties for CoCuLDH to achieve greater efficiency in ranitidine degradation in water. This is because lower hydroxyl bond content may promote extra OH⁻ in the solution, creating electrostatic repulsion with HSO₅⁻ from PMS (Duan et al. 2016), and hinder the contact between LDH and PMS. Figure 1 c and d show pore size distribution curve for CoFeLDH and CoCuLDH, respectively. The mean pore diameter of CoFeLDH and CoCuLDH were similar while pore volume of CoFeLDH was double compared to the CoCuLDH. BET surface area of CoFeLDH and CoCuLDH were 52 m^2^/gm and 25 m^2^/gm, respectively as shown in Fig. 1e and f.

**Fig. 2:**
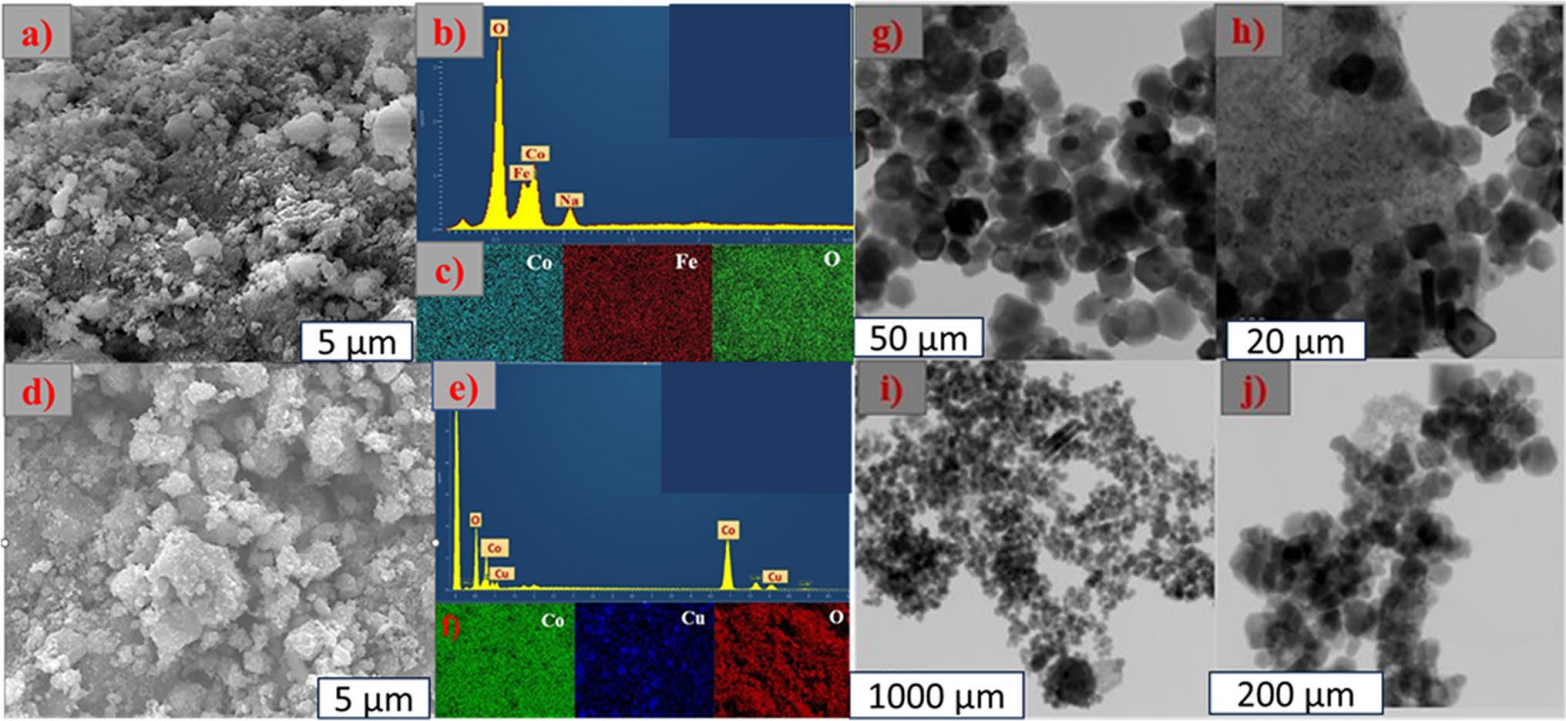
(**a**) SEM image of CoFeLDH, (**b**) EDS image of CoFeLDH, (**c**) EDS mapping images of CoFeLDH, (**d**) SEM image of CoCuLDH, (**e**) EDS image of CoCuLDH, (**f**) EDS mapping images of CoCuLDH, (**g**) TEM of CoFeLDH, (**h**) TEM of CoCuLDH, (**i**) STEM of CoFeLDH, and (**j**) STEM of CoCuLDH

The structural and morphological characteristics of CoFeLDH and CoCuLDH were studied using SEM. CoFeLDH exhibits a two-dimensional layered bumpy structure with a dense stacking effect (Fig. 2a), while CoCuLDH displayed a bumpy structure with agglomerated nanosheets (Fig. 2d). Both CoFeLDH and CoCuLDH had a polycrystalline structure, which renders them capable of being successful nanocatalysts. The EDS results of CoFeLDH and CoCuLDH are displayed in Fig. 2b and e, respectively. The mapping results (Fig. 2c and f) showed that Co, Fe, and Cu were uniformly distributed. TEM (Fig. 2g and h) and STEM (Fig. 2i and j) of CoFeLDH and CoCuLDH show a perfect hexagonal morphology.

### Analysis of CoFeLDH and CoCuLDH with PMS system in degradation of ranitidine

#### Influence of catalyst loading

**Fig. 3:**
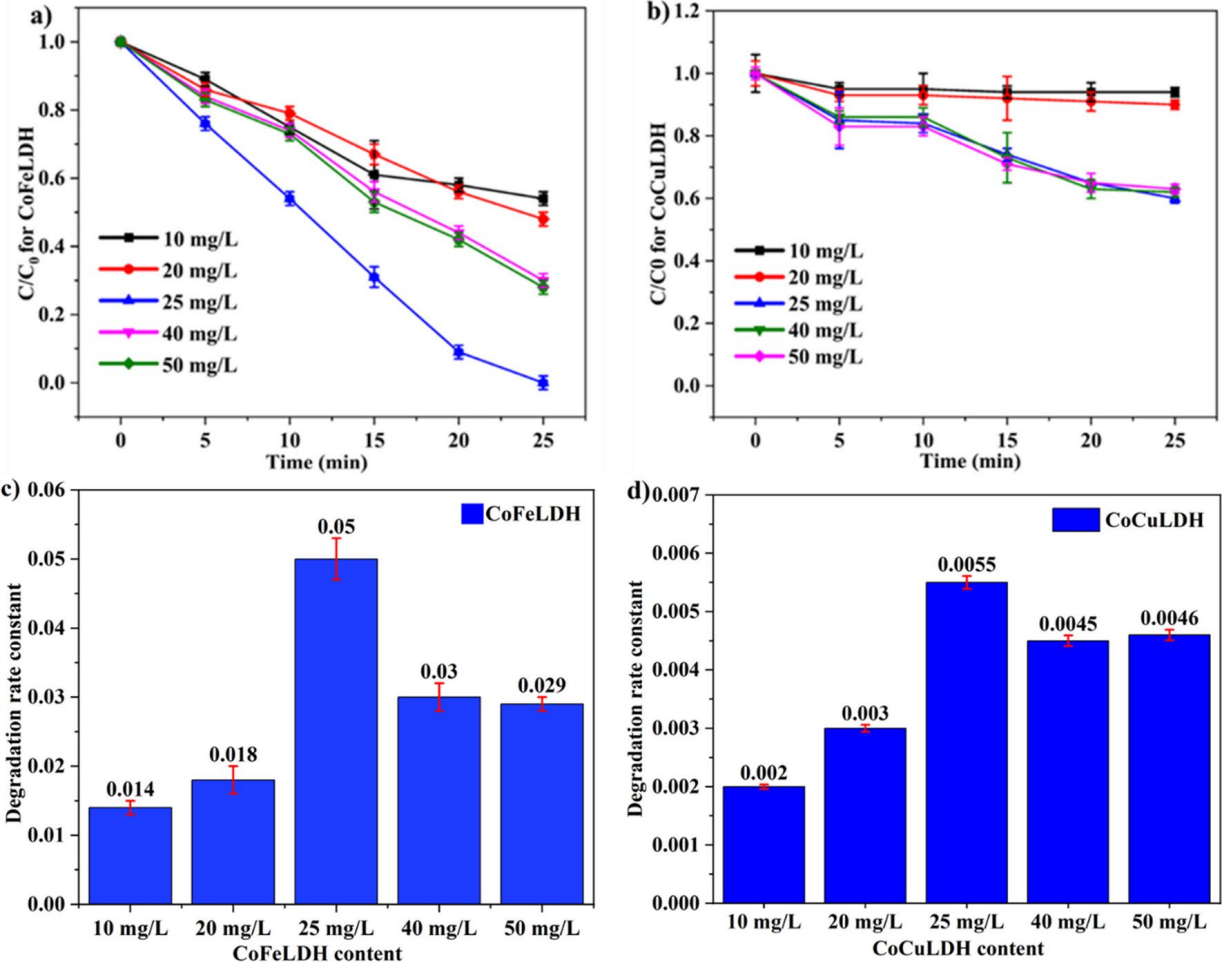
Influence of catalyst dosage (10–50 mg/L) on degradation vs reaction time curve for (**a**) CoFeLDH and (**b**) CoCuLDH, and degradation rate constant of ranitidine for (**c**) CoFeLDH and (**d**) CoCuLDH. Experimental conditions: 20 mg/L of PMS, ranitidine concentration of 5 mg/L, and room temperature (20 ± 2 °C). The standard deviation among triplicate samples remained less than 5%

PMS acts as an oxidant in AOPs, and LDH is considered an excellent catalyst to accelerate the activation capacity of the oxidant. Understanding the influence of the catalyst and oxidant on micropollutant degradation is crucial for optimizing their amounts with respect to cost. A 25 mg/L LDH dosage provided the best degradation performance (Fig. 3a), making it the optimum dosage for a 20 mg/L PMS dosage. The highest degradation of ranitidine (100%) occurred with a 25 mg/L LDH content, while the lowest degradation (61%) occurred with a 10 mg/L LDH content. However, around 20 mg/L LDH began to provide satisfactory degradation (85%). Similar findings were reported for CoFeLDH, where an LDH content of 50 mg/L started to provide degradation of ciprofloxacin above 80% at a PMS dosage of 40 mg/L and a ciprofloxacin concentration of 20 mg/L (Yang et al. 2021). The reason for using more LDH and PMS for degradation in previous literature may be due to the high initial concentration of ciprofloxacin. On the other hand, CoCuLDH showed only 40% degradation of ranitidine under similar conditions (Fig. 3b). CoCuLDH also followed the same trend, with degradation increasing as catalyst dosage increased. As LDH is used as a nanocatalyst for promoting the oxidation of PMS, more LDH content ensures more oxidation and more degradation. Moreover, an increase in LDH content provides more active sites in the solution and increases degradation (Ma et al. 2019). From Fig. 3a, degradation efficiency slightly decreased beyond a 25 mg/L LDH content. This may be attributed to the fact that an excess amount of catalyst may cause collisions and shear stress rather than promoting the catalytic effect, which may reduce degradation efficiency. The effect of LDH becomes more prominent when measuring the degradation rate constant, as shown in Fig. 3c and d. The reaction rate constants were found to be 0.03 (R^2^ = 0.89), 0.03 (R^2^ = 0.96), 0.11 (R^2^ = 0.99), 0.05 (R^2^ = 0.97), and 0.05 (R^2^ = 0.97) per min for 10, 20, 25, 40, and 50 mg/L LDH respectively. The results suggest that 25 mg/L LDH worked best for ranitidine degradation, and the influence of 40 and 50 mg/L LDH dosages was almost similar. A certain amount of PMS dosage was oxidized by optimized LDH content, and the rest of the LDH might be involved in side effects (Chen et al. 2019).

#### Influence of PMS dosage

**Fig. 4:**
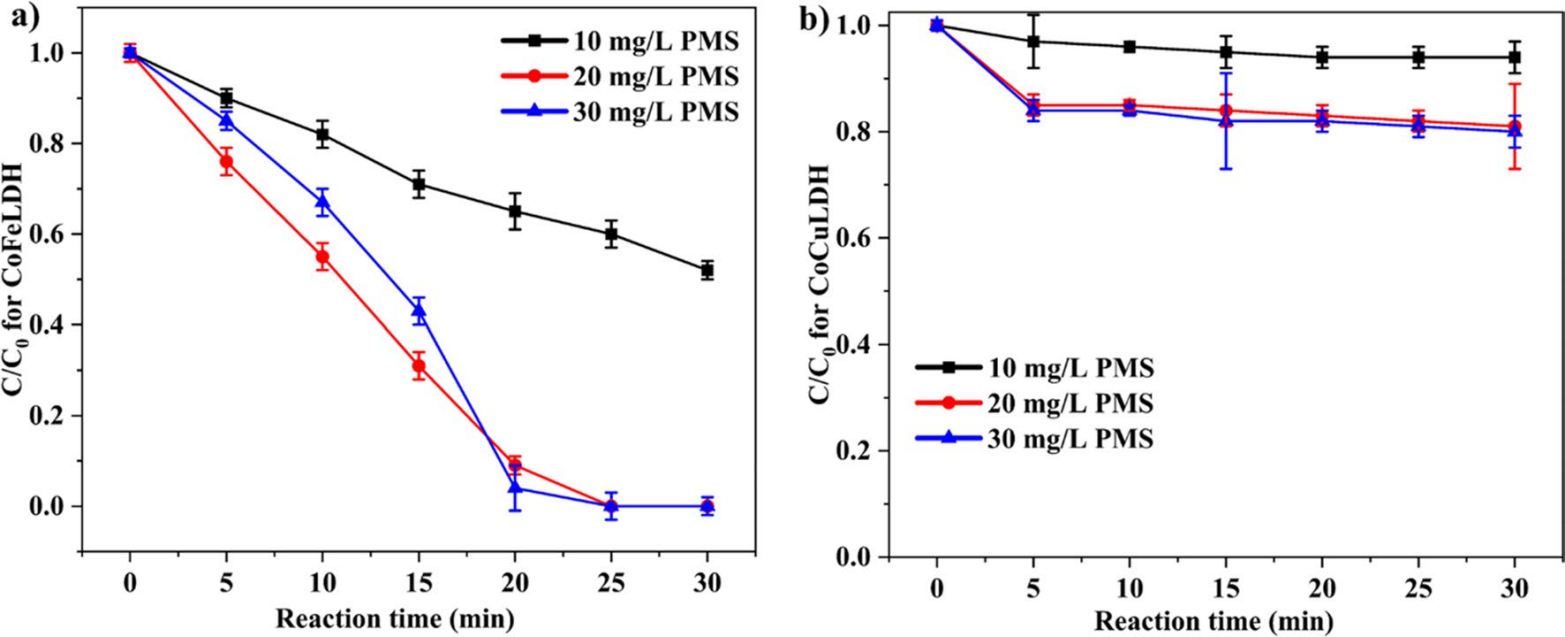
Influence of PMS dosage (10–30 mg/L) on the degradation rate constant of ranitidine for (**a**) CoFeLDH, and (**b**) CoCuLDH. Experimental condition: LDH 25 mg/L, ranitidine concentration of 5 mg/L, and room temperature (20 ± 2 °C). The standard deviation among triplicate samples remained less than 5%

Degradation of ranitidine increased with the increase of PMS dosage from 10 to 30 mg/L when the LDH content was fixed at 25 mg/L (Fig. 4a). Similar findings were observed for CoFeLDH during the degradation of ciprofloxacin, where degradation increased for PMS dosages ranging from 30 to 60 mg/L at 50 mg/L LDH (Yang et al. 2021). This may be because PMS, being an oxidant in this context, helped to increase the efficiency of degradation. This PMS requirement varied with the change in LDH content (Lu et al. 2019). In contrast, excessive PMS can cause self-activation, which requires sulfate radicals and hence reduces degradation (Chen et al. 2019). The results suggest that a 20 mg/L PMS dosage was optimal for a 25 mg/L LDH content, where 100% ranitidine was degraded. On the other hand, although the effect of PMS was similar for CoCuLDH as for CoFeLDH, ranitidine degradation was only 40% (Fig. 4b) at 20 min. Probably, 25 mg/L LDH and 20 mg/L PMS dosage were not sufficient for ranitidine degradation, for which the degradation was very low. This degradation efficiency increased to 70% after 60 min. Similar findings were observed for lomefloxacin degradation by CoCuLDH, where 40 mg/L LDH and 150 mg/L PMS degraded 96% of lomefloxacin (Guo et al. 2020), indicating that low LDH and PMS dosages were responsible for this slow rate and low degradation efficiency. In principle, CoFeLDH performed better for ranitidine degradation than CoCuLDH.

#### Influence of pH

**Fig. 5:**
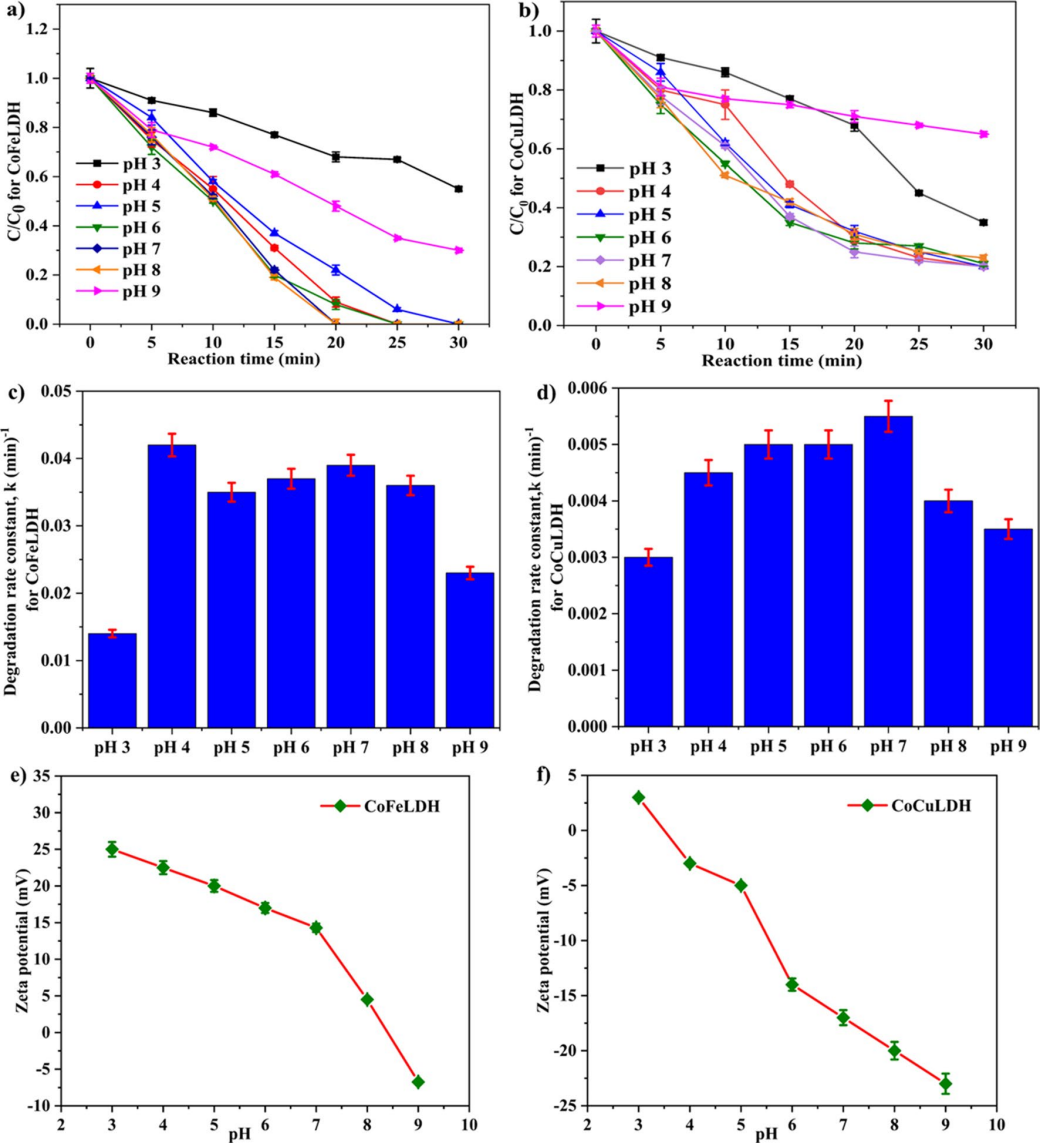
Influence of pH on ranitidine degradation for (**a**) CoFeLDH and (**b**) CoCuLDH, and on degradation rate constant of ranitidine for (**c**) CoFeLDH and (**d**) CoCuLDH, (**e**) zeta potential at different pH for CoFeLDH, and (**f**) zeta potential at different pH for CoCuLDH. Experimental condition: catalyst dosage of 25 mg/L, PMS of 20 mg/L, ranitidine concentration of 5 mg/L, pH unadjusted, and room temperature (20 ± 2 °C). The standard deviation among triplicate samples remained less than 5%

The effect of pH on the degradation of ranitidine is shown in Fig. 5a, demonstrating that ranitidine degradation is more effective for pH levels ranging from 4 to 8. Similar results were found for ranitidine degradation by CoAlLDH, nitrobenzene degradation by the CoFeLDH nanosheets/PMS system, and CuCoFeLDH (Lu et al. 2019). This can be attributed to the fact that LDH/PMS system is a radical-based catalytic oxidation process, where the generation of more effective radicals accelerates the oxidation process. Sulfate radicals with longer lifespans are produced in acidic conditions, which can positively impact degradation efficiency in the acidic pH range. At higher pH levels, self-dissociation of PMS occurs, and therefore electrostatic repulsion between PMS and LDH reduces degradation efficiency (Ye et al. 2020). Moreover, CoFeLDH has positive zeta potential at neutral pH. The pH range of 4 to 8 provided the maximum efficiency of ranitidine degradation, while pH 3 and pH 9 yielded the lowest degradation efficiency. This phenomenon can be attributed to the fact that CoFeLDH exhibits a positive charge at pH levels of 4 to 8, which promotes the adsorption of negatively charged micropollutants (Oliveira et al. 2019; Panplado et al. 2019). Moreover, LDH possesses a positive charge under acidic conditions, creating favorable conditions for HSO_5_^−^ to be attracted to the catalyst, thereby facilitating the degradation process. In contrast, an excessive amount of H^+^ may play a negative role in the activation of PMS by creating hydrogen bonds with the O–O group of HSO_5_^−^ (Zhang et al. 2013). This could inhibit the contact between positively charged catalyst surface and HSO_5_^−^, which also carries a positive charge, resulting in insignificant outcomes at pH 3. At pH 3, the surface charge of LDH becomes more positive, which may create electrostatic repulsion with positively charged pollutants. LDH may undergo self-degradation at low pH values [38]. At pH levels greater than 9, CoFeLDH carries a negative charge and can only absorb positively charged micropollutants (Ghemit et al. 2018).

Figure 5b represents the degradation vs. time graph for ranitidine degradation by CoCuLDH. CoCuLDH and CoFeLDH exhibit almost similar characteristics under the effect of pH. The key difference is that CoFeLDH performs better than CoCuLDH under all pH conditions, except similar degradation was noticed at alkaline solution. The degradation of ranitidine was around 70% at pH 4 to 8 and 60% at pH 9 for CoCuLDH, while the efficiency of ranitidine was 100% at pH 4 to 8 and only 63% at pH 9 for CoFeLDH. This phenomenon can be explained in terms of negative zeta potential of CoCuLDH. CoCuLDH has negative zeta potential of − 17 mV and it may create electrostatic repulsion with PMS, slowing the rate of LDH-based catalytic oxidation of PMS. The lower degradation under alkaline conditions may be due to the presence of Fe. Fe present in CoFeLDH may create hydroxide precipitates, which could inhibit the generation of Fe^2+^ as well as SO_4_^•−^ radicals [40], thereby reducing the degradation of ranitidine at pH 9 for CoFeLDH.

From Fig. 5c, the highest degradation rate constant was achieved for pH 4, suggesting faster degradation at pH 4 and pH 7. The value of the rate constant showed a more accurate effect of pH on degradation, showing that degradation of ranitidine performs better within the pH range of 4 to 8. PMS exists as HSO_5_^−^ in solution for pH ranging from 4 to 8 and converts to SO_5_^2−^ at pH 9, thereby preventing the generation of SO_4_^•−^ radicals. From Fig. 5d, the highest degradation rate constant was achieved at pH 7, and the second-highest rate constant observed at pH 4 and 6. The favorable pH range for ranitidine degradation by CoCuLDH was 4 to 7. The degradation rate constant of CoFeLDH was 10 times higher than the rate constant of CoCuLDH. This phenomenon can be explained from surface zeta potential of CoFeLDH and CoCuLDH. Figure 5 e and f show surface zeta potential values of CoFeLDH and CoCuLDH powder respectively at pH level 3 to 9. CoFeLDH has surface zeta potential of 15 mV at pH 7 which is favorable for the catalytic oxidation of PMS. In contrast, CoCuLDH has surface zeta potential of − 17 mV which may create electrostatic repulsion with PMS at pH 7. The surface charge properties of LDH nanomaterials are greatly influenced by the solution’s pH. A solution pH lower than 7 results in a positive charge on the material’s surface, ensuring more adsorption of HSO_5_^−^ from PMS and more reaction taking place. In contrary, a solution pH greater than 7 results in a negative surface charge on particles, creating electrostatic repulsion for negative ions from PMS. Thus, CoFeLDH performed well compared to the CoCuLDH.

#### Identification of reactive oxygen species (ROS)

The heterogenous catalytic system for PMS activation in AOPs is influenced by the production of ROS or radicals. The contribution of radicals to catalytic performance can be understood by introducing retarders. TBA and methanol were used as reaction retarders for both sulfate (SO_4_^•−^) and hydroxyl (^•^OH) radicals. Although methanol reacts at almost the same rate with ^•^OH and SO_4_^•−^ radicals, TBA reacts faster with ^•^OH radicals than with SO_4_^•−^ radicals. The influence of TBA and methanol appears almost similar, except for 75 mM TBA in CoFeLDH system (Fig. S1a). Similar findings were found for the CuCoFe LDH/PMS system (Lu et al. 2019), suggesting a greater contribution of ^•^OH radicals and SO_4_^•−^ radicals simultaneously. Notably, despite injecting 75 mM TBA, 81.9% degradation of ranitidine was achieved (Fig. S1b), while the maximum inhibition achieved by methanol was 50%. A comparable result was observed for the CoAlLDH membrane, where 78.9% degradation occurred with 90 mM TBA (Asif et al. 2022). This may suggest that SO_4_^•−^ radicals were the primary reactive species influencing the reaction. The dominancy of SO_4_ radicals has been reported for micropollutants degradation in typical CoFe and CuCoFeLDH/PMS systems (Ma et al. 2021). P-Benzoquinone was used as an oxygen radical quencher, and it successfully retarded the reaction. After the addition of 2 mM p-benzoquinone, the degradation efficiency of ranitidine dropped to only 15.8%, confirming the presence and contribution of oxygen radicals. L-histidine (10 mM) was used as a retarder for singlet oxygen, and a degradation efficiency of 25% was observed. Similar results were achieved for the degradation of tetracycline (60%) by CoFeLaLDH after the addition of 10 mM L-histidine. Concentration of radical quencher was selected according to the previous literature. Singlet oxygen is a part of the non-radical mode, implying that both radical and non-radical pathways significantly contribute to ranitidine degradation by CoFeLDH.

From Fig. S1b, CoCuLDH system for ranitidine degradation shows that the highest degradation (80%) occurred with the addition of 2 mM p-benzoquinone. This indicates a lower contribution of oxygen (O_2_^•−^) radicals in ranitidine degradation in the CoCuLDH system. Similar results were found for ranitidine degradation (86%) in the CoAlLDHm/PMS system (Asif et al. 2022) and for lomefloxacin degradation (85%) in the CoCuLDH system (Guo et al. 2020). The lowest degradation occurred with 50 mM methanol and 2 mM L-histidine, with degradation efficiency of 35% and 30%, respectively. This implies that ^•^OH radicals and singlet oxygen (^1^O_2_) contribute to ranitidine degradation by CoCuLDH. The contribution of ^•^OH radical and singlet (^1^O_2_) has been found for ranitidine degradation by the CoAlLDHm/PMS system (Asif et al. 2022). The inhibiting effect of 75 mM TBA was only 15%, indicating the dominance of SO_4_^•−^ radicals in ranitidine degradation by the CoCuLDH system.

#### Exploration of catalytic mechanism to understand the role of transition metal

**Fig. 6:**
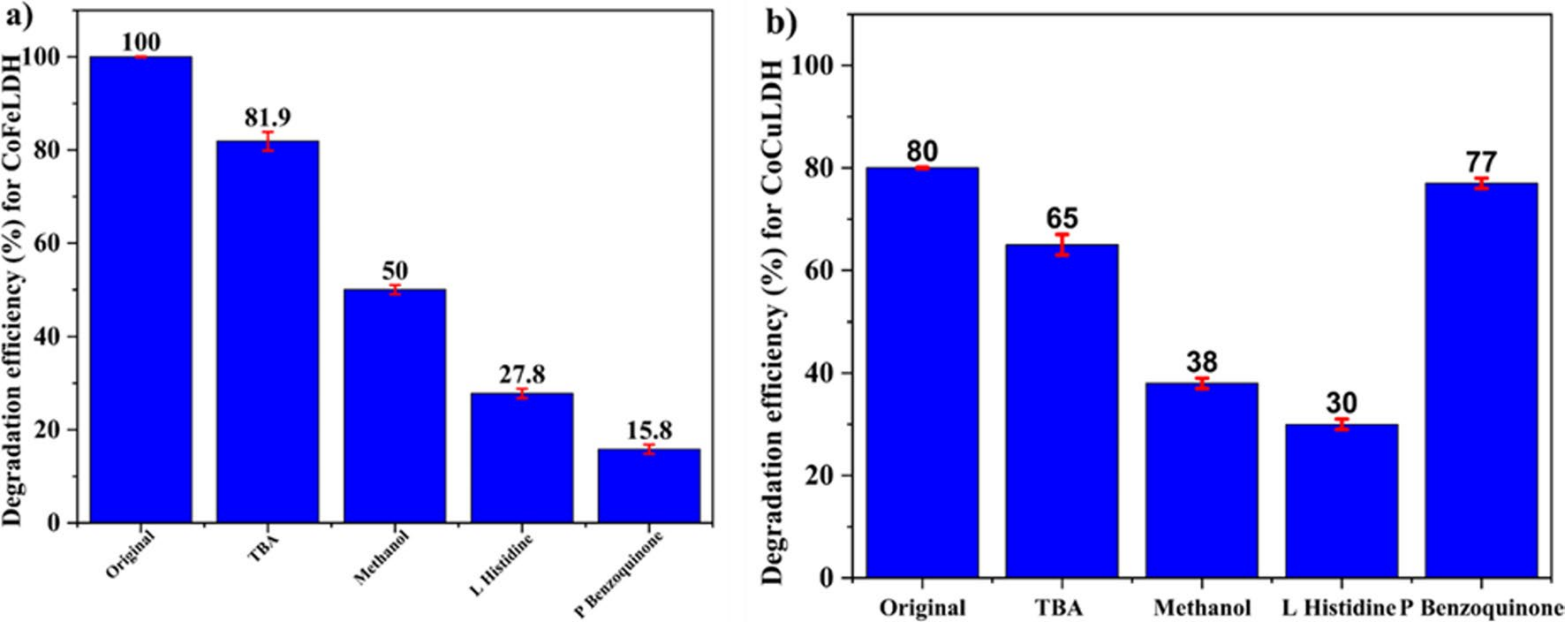
Effect of different radical quenchers on the degradation of ranitidine for (**a**) CoFeLDH and (**b**) CoCuLDH. Experimental condition: catalyst dosage of 25 mg/L, PMS of 20 mg/L, ranitidine concentration of 5 mg/L, pH unadjusted, and room temperature (20 ± 2 °C). The standard deviation among triplicate samples remained less than 5%

Understanding the catalytic mechanism is crucial for comprehending the reactions among the catalyst, oxidant, and target pollutants. Figure 6 a and b clearly show the contributions of SO_4_^•−^, ^•^OH, and O_2_^•−^ radicals, and singlet oxygen in ranitidine degradation for two different transition metal–based LDH/PMS systems. This test clarifies how the role of different radicals varies with the change in the transition metal of the LDH catalyst. TBA, mainly an OH radical quencher, reduced degradation by 20% for CoFeLDH and 15% for CoCuLDH, indicating a lower impact of ^•^OH radicals compared to SO_4_^•−^ radicals in ranitidine degradation. Methanol, acting as a quencher for both SO_4_^•−^ and ^•^OH radicals, reduced degradation by 50% and 42% for CoFeLDH and CoCuLDH, respectively. This suggests that while the SO_4_^•−^ radical plays dominant role in ranitidine degradation, the role of the ^•^OH radical is not negligible. Singlet ^1^O_2_ is not generated by redox reactions between transition metals and HSO_5_^−^ through electron transfer (Zhu et al. 2018b), and is therefore referred to as a non-radical mode. It can be produced in two ways: by the intercalated CO_3_^2−^ anion or by ^•^OH radicals. CO_3_^2−^ forms carbonyl groups (CO), and singlet ^1^O_2_ is created by oxidation (Duan et al. 2016). An excess of ^•^OH radicals can form singlet ^1^O_2_ (Zhu et al. 2018a) as per the following reaction 1:

1 $${2\text{OH}}^{\bullet }\to {\frac{1}{2}\text{O}}_{2 }+{\text{H}}_{2 }\text{O}$$

Singlet ^1^O_2_ quencher L-histidine reduced ranitidine degradation by 27.8% and 30% for CoFeLDH and CoCuLDH, respectively. Since singlet oxygen is a non-radical pathway of ranitidine degradation, its quencher, L-histidine, shows similar reductions for both LDHs, as the non-radical pathway does not depend on transition metals in LDH. In contrast, O_2_^•−^ radical quencher p-benzoquinone reduced ranitidine degradation by 85% for CoFeLDH but only 3% for CoCuLDH. The contribution of O_2_^•−^ radicals indicates that they play a significant role in ranitidine degradation by the CoFeLDH/PMS system. This may be the reason behind the large variation in optimal LDH and PMS dosages for CoFeLDH and CoCuLDH. When ranitidine is degraded in the CoFeLDH/PMS system, SO_4_^•−^, ^•^OH, and O_2_^•−^ radicals all participate in the degradation process. This participation results in a reduced dosage of LDH and PMS for degradation. In contrast, during the degradation of ranitidine in the CoCuLDH/PMS system, only SO_4_^•−^ and ^•^OH radicals perform the full radical pathway for degradation, and no O_2_^•−^ radical participate, necessitating a large amount of LDH and PMS dosage for ranitidine degradation in the CoCuLDH/PMS system, for which degradation efficiency is low. Similar findings were assessed for lomefloxacin degradation in CoCuLDH/PMS system by previous researcher, where SO_4_^•−^ and ^•^OH radicals, and singlet ^1^O_2_ were the main reaction species (Guo et al. 2020). A question may arise regarding the fate of the O_2_^•−^ radicals in the CoCuLDH system, namely whether they would not be produced or would not participate in the degradation process. SO_4_^•−^, ^•^OH, and O_2_^•−^ radicals are produced through the following reactions (Eqs. 2–11):

2 $${\text{Co}}^{2+}+ {\text{H}}_{2}\text{O}\to {\text{CoOH}}^{+}+ {\text{H}}^{+}$$

3 $${\text{Co}(\text{OH})}_{2}+ {\text{H}}^{+} \to {\text{CoOH}}^{+} {\text{H}}_{2}\text{O}$$

4 $${\text{CoOH}}^{+}+ {{\text{HSO}}_{5}}^{-} \to {\text{CoO}}^{+}+ {{\text{SO}}_{4}}^{\bullet -}+ {\text{H}}_{2}\text{O}$$

5 $${\text{Fe}}^{3+}+ {{\text{HSO}}_{5}}^{-}\to {\text{Fe}}^{2+}+{{\text{SO}}_{5}}^{\bullet -}+{\text{H}}^{+}$$

6 $${{2\text{SO}}_{5}}^{\bullet -}\to {{2\text{SO}}_{4}}^{2-}+{1\text{O}}_{2}$$

7 $${\text{Cu}}^{2+}+{{\text{HSO}}_{5}}^{-}\to {\text{Cu}}^{+}+{\text{OH}}^{\bullet }+{{\text{SO}}_{4}}^{2-}$$

8 $${{\text{SO}}_{4}}^{\bullet -}+{\text{OH}}^{-}\to {\text{OH}}^{\bullet }+{{\text{SO}}_{4}}^{2-}$$

9 $${{\text{SO}}_{4}}^{\bullet -}+{\text{H}}_{2}\text{O}\to {\text{OH}}^{\bullet }+{{\text{SO}}_{4}}^{2-}+{\text{H}}^{+}$$

10 $${{\text{OH}}^{.}}^{\bullet }+{\text{H}}_{2}{\text{O}}_{2}\to {{\text{HO}}_{2}}^{\bullet }+{\text{H}}_{2}\text{O}$$

11 $${{\text{HO}}_{2}}^{\bullet -}\to {\text{H}}^{+}+{{\text{O}}_{2}}^{\bullet -}$$

This oxygen radical may transform into singlet oxygen and participate in ranitidine degradation in the LDH/PMS system. Equation 12 is as follows:

12 $${{\text{O}}_{2}}^{\bullet -}+{\text{OH}}^{\bullet }\to {\text{OH}}^{-}+{1\text{O}}_{2}$$

The octahedral structure of LDH allows for reversible redox reactions involving cobalt (Co) and iron (Fe). Additionally, the mutual valence changes between Co(II) and Fe(III) facilitated the degradation of ranitidine during the catalytic process (Wang et al. 2023). The oxidation of Co^2+^ contributed to enhancing the electron density surrounding Fe^3+^. The interaction between cobalt (Co) and iron (Fe) in CoFeLDH enhances electron transfer (Wang et al. 2021), boosting its efficiency and stability in activating peroxymonosulfate (PMS) and producing reactive species like SO_4_^•−^ and ^•^OH radicals, and singlet ^1^O_2_. This accelerates the degradation of organic pollutants, ensuring effective remediation. In addition, for CoFeLDH/PMS system, the solution pH may vary with time, and CoFeLDH may form Fe(OH)_3_ precipitates at high pH, which could inhibit the formation of singlet ^1^O_2_ from O_2_^•−^ radicals. These radicals can participate in ranitidine degradation with greater efficiency and promote the dominancy of the radical pathway of ranitidine degradation in the CoFeLDH/PMS system.

**Fig. 7:**
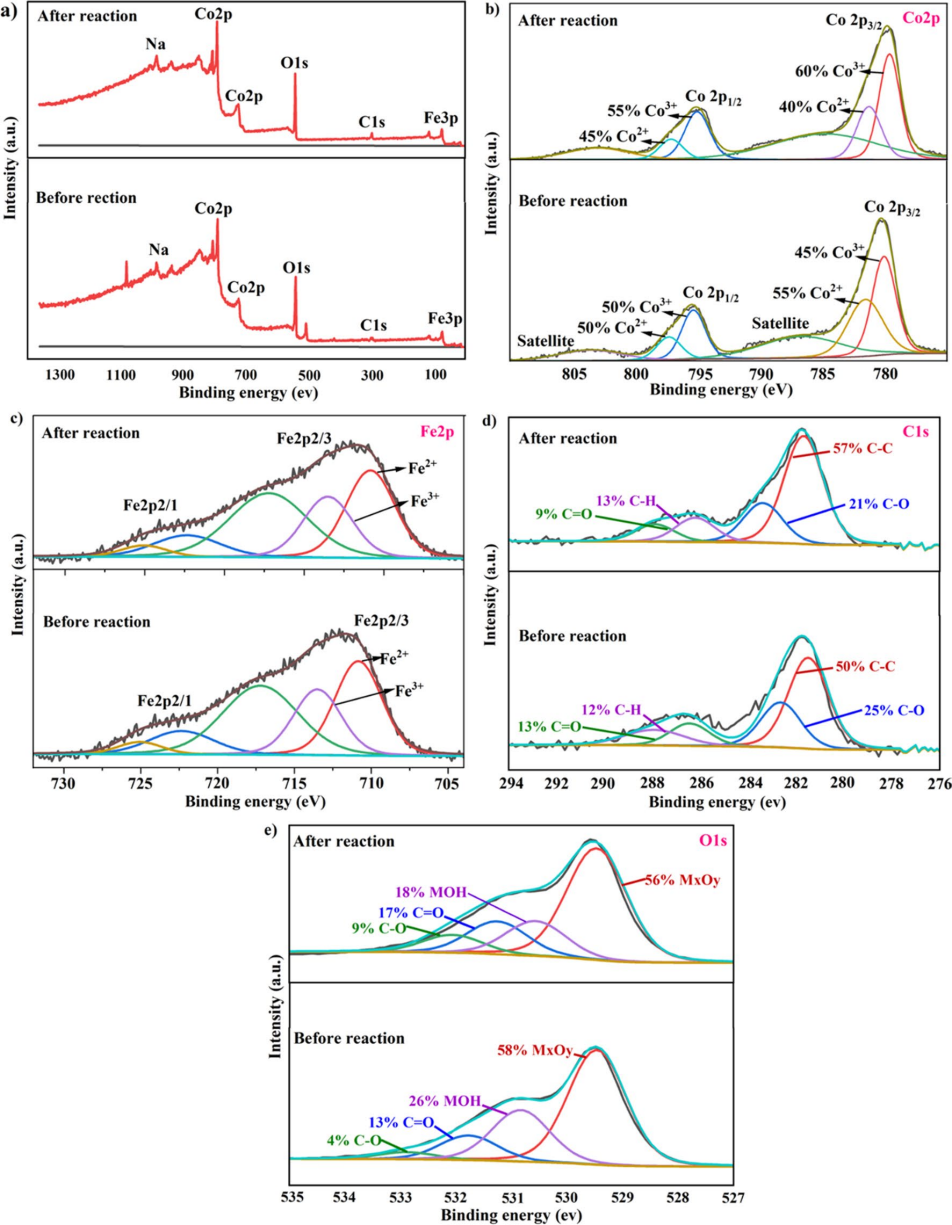
High-resolution XPS spectra of (**a**) XPS survey, (**b**) Co 2p, (c) Fe 2p, (**d**) C 1 s, and (**e**) O 1 s of CoFeLDH powder before and after reaction with PMS and ranitidine

XPS analysis was done to reveal the intermediate phase of the catalytic reaction between the CoFeLDH catalyst and PMS. XPS data of LDH catalyst before and after reaction exhibited the change in the valence state of transition metals Co and Fe after filtration of polluted water. It also proved the reusability of CoFeLDH powder. XPS survey, Co2p, O1s, and C1s spectra are shown in Fig. 7a–f, respectively. XPS survey spectra of before and after reaction demonstrate the presence of Co2p, O1s, C1s, and Fe2p in similar binding energy. For Co2p, two peaks are observed at 780 eV and 795 eV, indicating the presence of both Co^2+^ and Co^3+^ in different proportions. The peak at 795 eV corresponds to Co2p1/2, while the peak at 780 eV is associated with Co2p3/2. These peaks are characteristic of Co(OH)_2_ and Co_3_O_4_, which play distinct roles in PMS activation. In the before reaction, Co^2+^ (originating from Co(OH)_2_) constituted 55%, and Co^3+^ (from Co_3_O_4_) made up 45%, reflecting the + III oxidation state before PMS activation. After use, Co^2+^ decreased to 40%, while Co^3+^ increased to 60%, illustrating the transformation of Co^2+^ to Co^3+^ and the recycling of Co^3+^ to Co^2+^. Notably, the electron transfer reaction from Co^2+^ to PMS is faster than the recycling from Co^3+^ to Co^2+.^ In the Fe 2p spectrum of CoFeLDH (Fig. 7c), a slight shift toward lower binding energy is observed after the reaction. This indicates that the oxidation of Co^2^⁺ contributes to the electron cloud density of Fe^3^⁺.

Figure 7d shows XPS spectra of C1s orbital for the catalyst before and after the reaction. Four peaks were found for C–C, C-O, C-H, and C = O at a binding energy of 281.8, 284, 287.5, and 289.2 eV for fresh CoFeLDH powder. After the reaction, all four peaks shifted right, denoting binding energy of 281, 282.5, 285.8, and 287.5 eV. The percentage of C–C bond and C-O bond increased from 46 to 57% and 7 to 21%, respectively, after reaction and the percentages of C-H and C = O bond decreased from 30 to 13% and 16 to 9%, respectively, by catalytic degradation. Therefore, the formation and breakdown of the bonds were clearly visible through XPS analysis. The O1s spectra of after reaction displayed four characteristic peaks at binding energies of 529.5 eV, 531 eV, 532 eV, and 533 eV. The most prominent peak, accounting for 58% and representing metal oxide, was observed at 529.5 eV. The second-highest peak, indicative of metal hydroxide, appeared at 531 eV, comprising 26% of the spectrum. In the before rection, the metal oxide content was double that of metal hydroxide. However, after reaction, metal hydroxide reduced to 18%. Conversely, the C = O content in the after reaction increased to 17%, from 13% in the before reaction. The proportion of metal oxides remained consistent even after the PMS-based oxidation process through LDH. The decrease in metal hydroxide content in the after reaction suggests the formation of Co_3_O_4_ and Fe_3_O_4_ from Co(OH)_2_ and Fe(OH)_2_. In addition, the peaks of metal hydroxide, C = O, and C-O shifted toward lower binding energy slightly for after reaction compared to the peaks corresponding to the before reaction.

SO_4_^•−^ was considered dominant radicals for ranitidine degradation which was formed by the reaction between PMS and divalent transition metal of the catalyst surface. This SO_4_^•−^ radical reacted with water molecules and formed ^•^OH radical. Both SO_4_^•−^ and ^•^OH radicals proceeded to degrade micropollutants. Trivalent transition metals formed SO_5_^•−^ radicals instead of SO_4_^•−^ radicals which also had a contribution in micropollutants degradation. Standard reduction potential of Co^3+^/Co^2+^ is 1.81 V where reduction potential of HSO_5_^−^/SO_5_^•−^ is 1.1 V. For this reason, the conversion of Co^3+^ to Co^2+^ by HSO_5_^−^ is thermodynamically advantageous (Huang et al. 2017). In addition, the increase of Co^2+^ was favorable to continue the activation of PMS at further cycle.

### Effect of anions and humic acid to understand the environmental stability of CoFeLDH

**Fig. 8:**
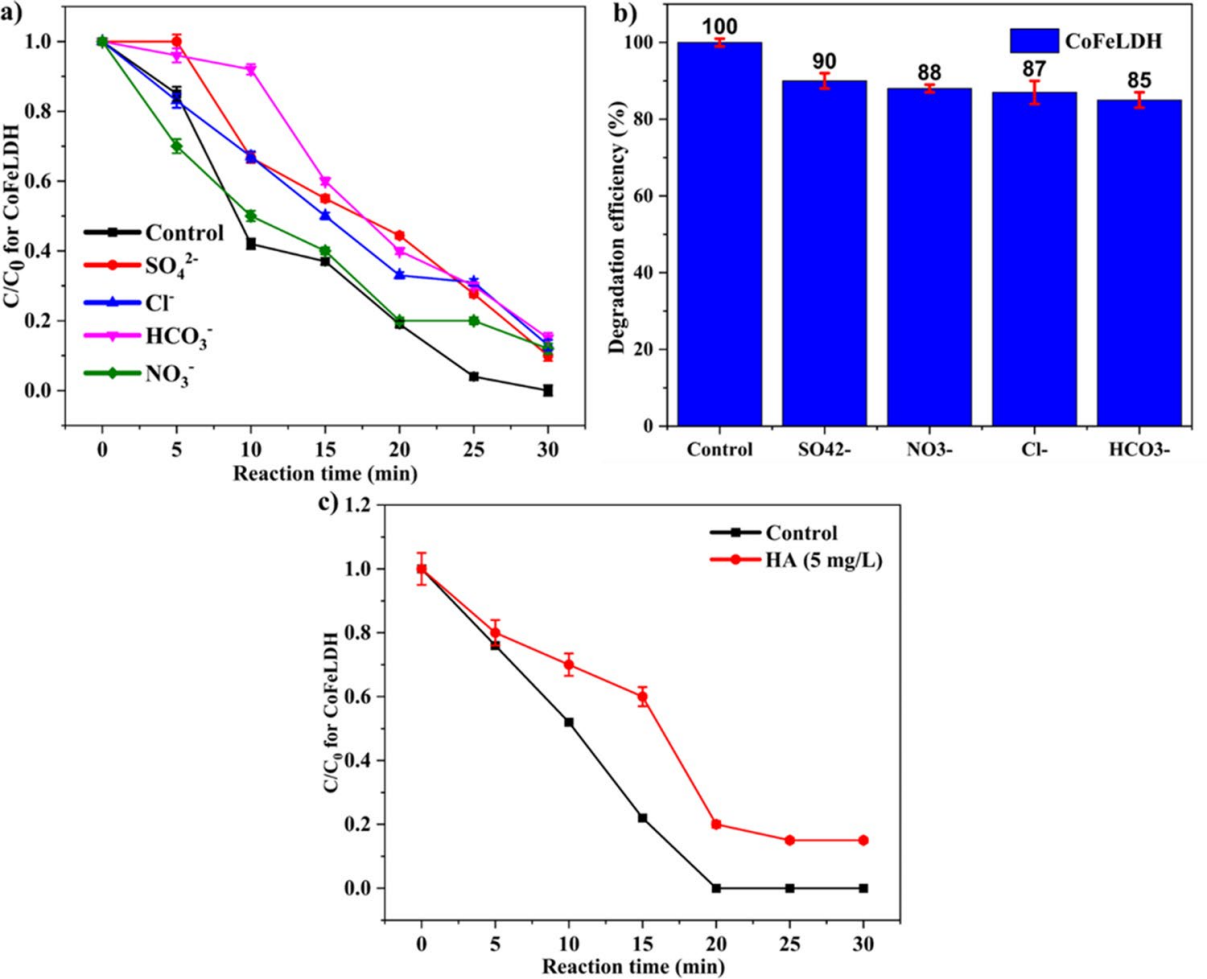
(**a**) Effect of different anions (5 mM) on the degradation of ranitidine vs time, (**b**) effect of different anions (5 mM) on degradation rate constant, and (**c**) effect of humic acid (5 mg/L) in CoFeLDH/PMS system. Experimental condition: 25 mg/L of LDH, 20 mg/L of PMS, ranitidine concentration of 5 mg/L, pH unadjusted, and room temperature (20 ± 2 °C). The standard deviation among triplicate samples remained less than 5%

The effect of anions on degradation efficiency is essential for understanding the ability of the catalyst to function in natural water. Natural water contains various anions such as HCO_3_^−^, SO_4_^2−^, NO_3_^−^, and Cl^−^, which can reduce the catalytic degradation efficiency to some extent. Therefore, it is crucial to illustrate the role of anions in the catalytic degradation in the LDH/PMS system. Four additional anions and humic acid (HA) were introduced during the degradation process, and the degradation efficiency of ranitidine was slightly reduced. The highest reduction in degradation was caused by HCO_3_^−^ (around 15%), while the lowest reductions in degradation were caused by SO_4_^2−^ (10%), NO_3_^−^ (12%), and Cl^−^ (13%) (Fig. 8a and b). Similar findings were observed for SO_4_^2−^ (20%), NO_3_^−^ (25%), and Cl^−^ (25%) in previous literature for ranitidine removal through CoAlLDH membrane (Asif et al. 2022). The inhibitory effect of HCO_3_^−^ is due to its alkalinity, which can hinder the acidic effect of PMS and inhibit the degradation process. The inhibitory effect of Cl^−^ can be attributed to the fact that Cl^−^ can react with free radicals and produce less reactive chloride radicals [47] (Eqs. 13 and 14). Moreover, Cl^−^ can form complexes with active sites and utilize reactive oxygen species [41]. HCO_3_^−^ and Cl^−^ showed a higher tendency to react with sulfate and hydroxyl radicals than NO_3_^–^, resulting in a lower inhibiting effect.

13 $${\text{Cl}}^{-}+{\text{OH}}^{\bullet }\to {\text{HOCl}}^{\bullet -}$$

14 $${\text{HOCl}}^{\bullet -}+{\text{H}}^{+}\to {{\text{Cl}}^{.}}^{\bullet }+{\text{H}}_{2}\text{O}$$

**Fig. 9:**
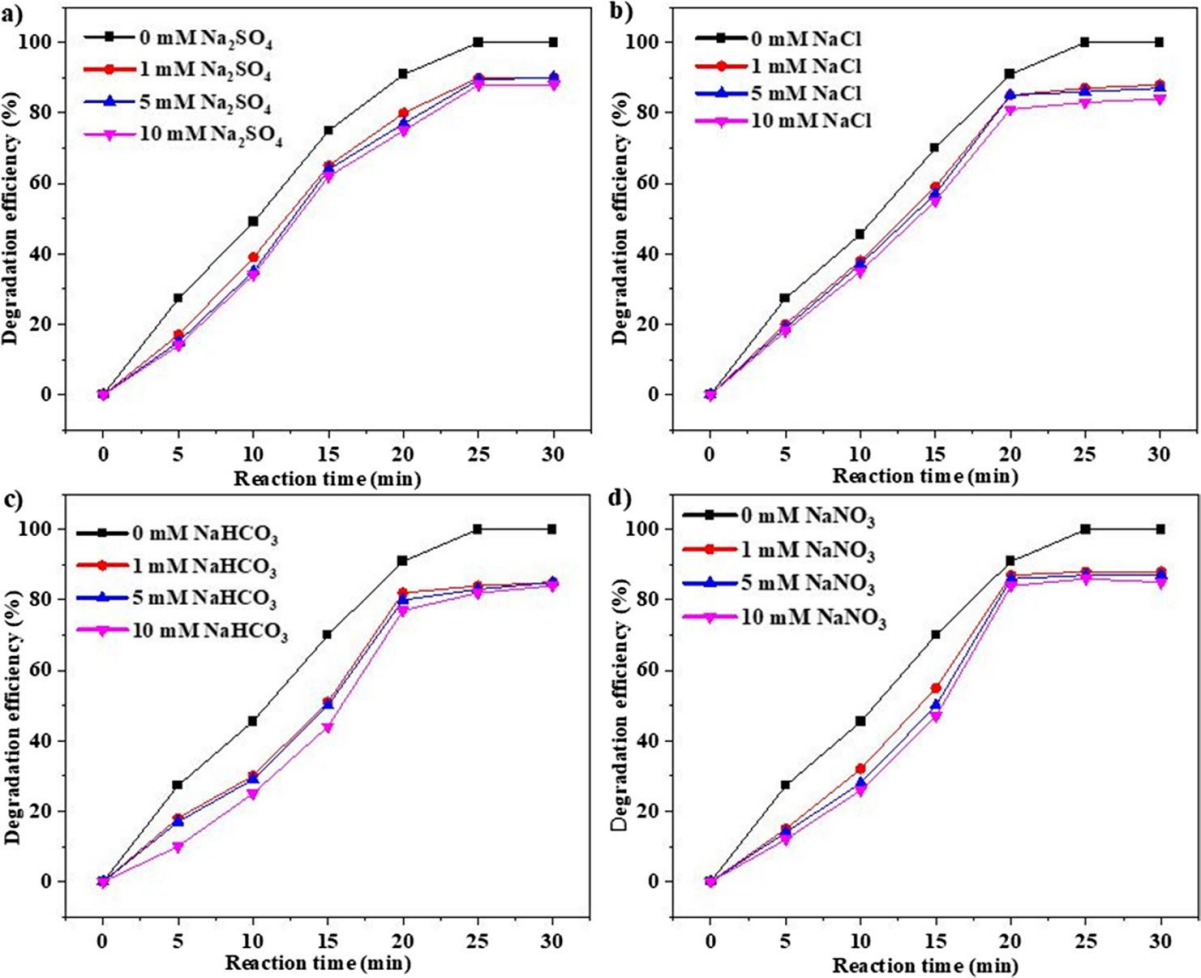
Effect of different anions with varying concentrations on the degradation of ranitidine by CoFeLDH catalysts (**a**) Na_2_SO_4_ (1, 5, and 10 mM), (**b**) NaCl (1, 5, and 10 mM), (**c**) NaHCO_3_ (1, 5, and 10 mM), and (**d**) NaNO_3_ (1, 5, and 10 mM). Experimental condition: 25 mg/L of LDH, 20 mg/L of PMS, ranitidine concentration of 5 mg/L, pH unadjusted, and room temperature (20 ± 2 °C). The standard deviation among triplicate samples remained less than 5%

Figure 8c shows the effect of humic acid on ranitidine degradation. The addition of humic acid reduced the degradation of ranitidine by up to 20%. This reduction can be attributed to the fact that humic acid present in wastewater can be adsorbed onto the surface of the LDH, consequently reducing the active area of the LDH that participates in the reaction (Lu et al. 2019). Moreover, humic acid can act as a radical scavenger as humic acid and OH radicals have a reaction rate constant of 1 × 10^8^ ∼ 2 × 10^8^ M^−1^·s^−1^ (Westerhoff et al. 2007). However, the reduction in degradation is not very significant because the presence of Fe in CoFeLDH may circulate due to the presence of organic substances, which can aid in the degradation process. From a structural perspective, humic acid has a complex configuration with functional groups such as aromatic rings, carbonyl groups, phenolic hydroxyl groups, and ethers. It has been demonstrated that a small amount of phenolic compounds and terpenoids in humic acid can generate free radicals by prompting the decomposition of PMS (Walton 2003). As a result, both the reaction with free radicals and the generation of free radicals occur simultaneously due to the presence of humic acid during ranitidine degradation in the CoFeLDH/PMS system. The anion concentrations were varied between 1 mM, 5 mM, and 10 mM in Fig. 9a–d, with negligible differences in the results. These results ensure the negligible effect of anion concentration on ranitidine removal.

### Reusability and leaching tests of CoFeLDH

**Fig. 10:**
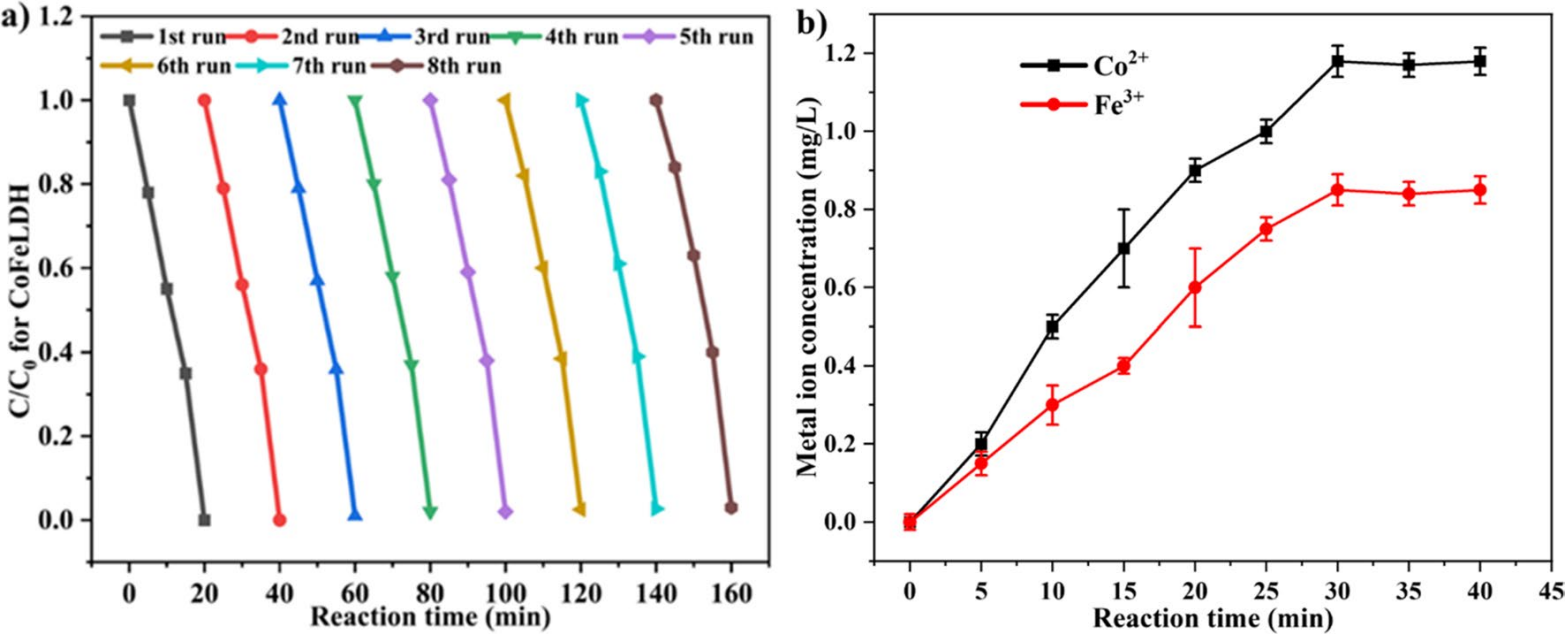
(**a**) The cycle stability test of CoFeLDH and (**b**) concentration of leaching metal ions during the degradation process for CoFeLDH. The standard deviation among triplicate samples remained less than 5%

The reusability test is crucial for the practical application of heterogeneous catalysts in AOPs. Four runs were conducted to assess the reusability of CoFeLDH catalyst. The degradation efficiency of ranitidine decreased from 100 to 97% after 8 runs of 160 min for CoFeLDH (Fig. 10a). The first and second runs did not affect degradation efficiency. Similar findings were observed for ciprofloxacin degradation by CoFeLDH, where the degradation efficiency reduced from 87 to 76% after 3 runs (Yang et al. 2021). This slight decrease may be due to the loss of catalyst during recycling, and the adsorption of pollutants on the LDH surface. This phenomenon can be explained by the leaching experiments conducted with CoFeLDH. The metal leaching test of CoFeLDH revealed the leaching of 1.2 mg/L Co^2+^ and 0.85 mg/L Fe^3+^ (Fig. 10b). Similar findings were achieved for ranitidine degradation by CoAlLDH membrane where a metal leaching of Co^2+^ was 23.14 µg/L in the membrane permeate (Asif et al. 2022) and negligible leaching of Fe^3+^ was found in MgCuFeLDH (Zhu et al. 2019). Excessive leaching can collapse the layered structure of the catalyst and reduce the recycling capability of LDH (Yang et al. 2021). In addition, Co^2+^ might leach out after the reaction, posing risks to the environment and human beings. Therefore, it is essential to identify the amount of metal ion leaching in transition metal–based catalysts. The leaching value and recycling test of CoFeLDH ensured the proper stability and reusability of the system.

### Identification of ranitidine degradation pathway in CoFeLDH/PMS system

**Fig. 11:**
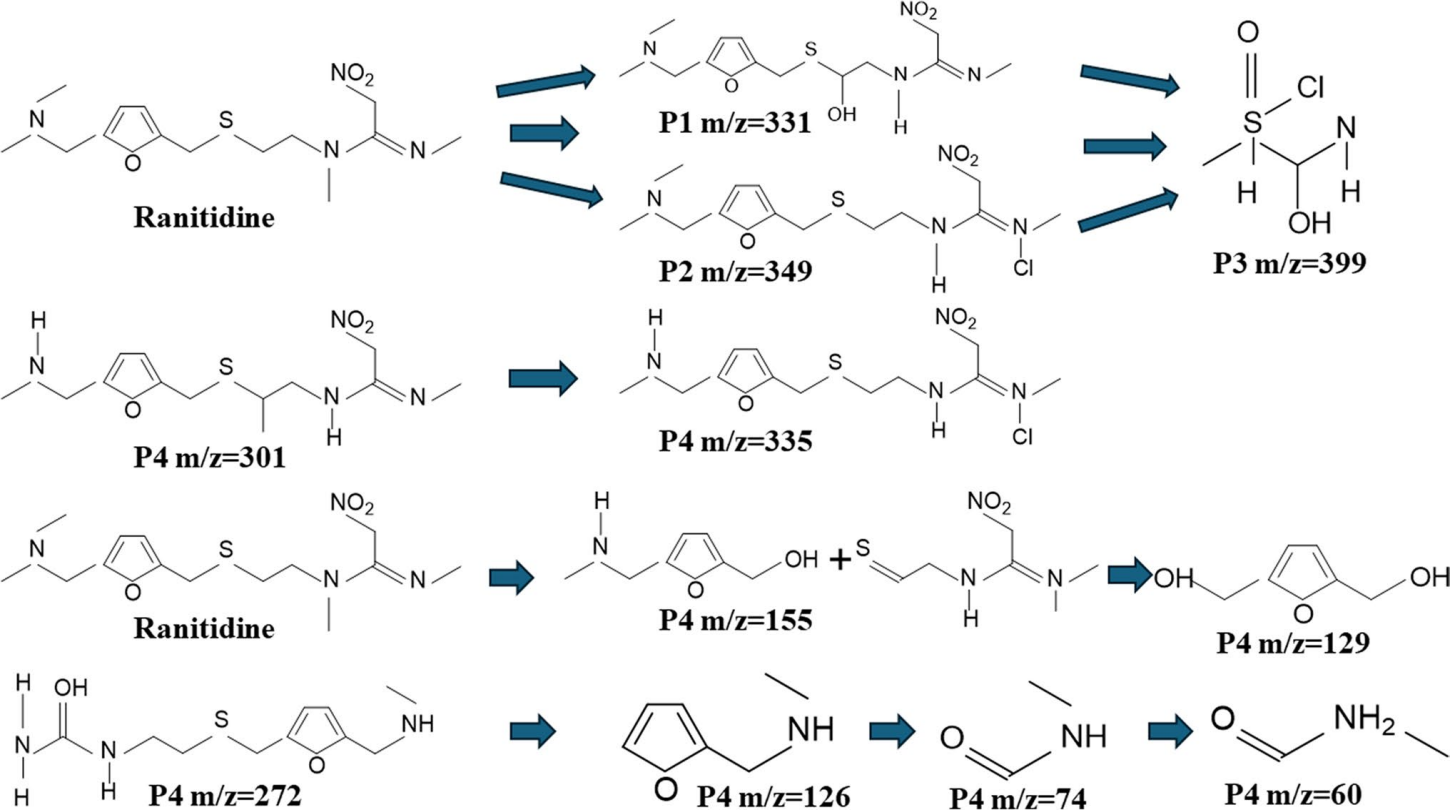
Proposed degradation pathway of ranitidine in the CoFeLDH/PMS system

In this study, intermediate products during ranitidine degradation were identified through LCMS, and mass spectrum of associated peaks was collected from the experiment. These associated peaks indicated the identification of intermediate products. SO_4_^•−^, O_2_^•−^, and ^•^OH radicals and singlet ^1^O_2_ were found to be the major oxidant species participating in the ranitidine degradation. As electrophilic radicals, SO_4_^•−^ and O_2_^•−^ radicals participated in the degradation through an electron transfer procedure (Oh et al. 2016; Zhou et al. 2017). ^•^OH radicals have multiple roles such as hydrogen abstraction, electrophilic addition, and indirect contribution to electron transfer to degrade ranitidine in CoFeLDH/ PMS system (De Laat et al. 2011). In this experiment, m/z 331, m/z 399, m/z 349, m/z 155, m/z 129, m/z 301, m/z 335, m/z 272, m/z 126, m/z 74, and m/z 60 were identified by positive ionization (Fig. 11). According to the intermediate products, degradation pathways of ranitidine and reaction procedures were predicted for ranitidine degradation. P3 (m/z 399) generated by hydroxylation and chlorine addition via P1 (m/z 331) and P2 (m/z 349) products. P5 (m/z 335) is produced through N-methylation via P4 (m/z 301). Following previous studies of ranitidine degradation, hydroxylation, chlorine addition, and N-methylation are common reactions for the degradation of ranitidine. Ranitidine contains an NH group, and this group can react with both SO_4_^•−^ and ^•^OH radicals to generate N-centered radicals (Yan et al. 2019). P12 (m/z 60) is produced through C-S and C-N bond cleavage and the intermediate products found were P7 (m/z 272), P8 (m/z 155), P9 (m/z 129), P10 (m/z 126), and P11 (m/z 74). As time proceeded, intermediate products vanished due to the oxidation capability of radicals, and ranitidine was eventually mineralized to CO_2_ and H_2_O.

## Conclusions

In this study, two types of transition metal–based LDH catalysts were successfully synthesized for the PMS-based advanced oxidation process to achieve effective removal of ranitidine. CoFeLDH showed superior catalytic capabilities (100% degradation in 20 min) compared to CoCuLDH in the degradation of ranitidine, with the only difference between them being a single transition metal. CoFeLDH possessed positive zeta potential of 15 mV while CoCuLDH had negative zeta potential of − 17 mV. Both LDH catalysts operated at different pH ranges and utilized various reactive species, with the CoFeLDH/PMS system exhibiting strong radical-based catalysis because of O_2_^•−^ radicals. Although SO_4_^•−^ played dominant role, the roles of OH^•^ and O_2_^•−^ radicals were also significant in the PMS-based CoFeLDH catalyst system for ranitidine degradation. Additionally, CoFeLDH catalyst showed better reusability (97% degradation even after 8 runs) and stability than traditional systems. XPS analysis also proved the reusability of this catalyst and the existence of divalent and trivalent metal cation with percentages at different phases. The findings of LCMS suggested a degradation pathway of ranitidine by CoFeLDH catalyst in PMS system. This catalyst has potential applications in wastewater treatment for the removal of different micropollutants besides ranitidine, and real water samples could be used for more diverse testing. Further optimization of operating conditions during membrane fabrication could enhance their potential. An assessment focusing on the detection of ROS through EPR analysis could offer a deeper understanding of the catalytic mechanism involved.

## Supplementary Information

Below is the link to the electronic supplementary material.

Supplementary file1 (DOCX 204 KB)

## Data Availability

The experimental data of this study is available upon request.
