# Supplementary material for: Ranitidine degradation in layered double hydroxide activated peroxymonosulfate system: impact of transition metal composition and reaction mechanisms
Source: Environ Sci Pollut Res Int. 2024 Jul 15;32(49):28343–60. doi: 10.1007/s11356-024-34331-5 (PMC12711975; doi:10.1007/s11356-024-34331-5)
Supplement: Supplementary file 1 — Supplementary file1 (DOCX 204 KB) [file 11356_2024_34331_MOESM1_ESM.docx]

**Supplementary Figure**

**
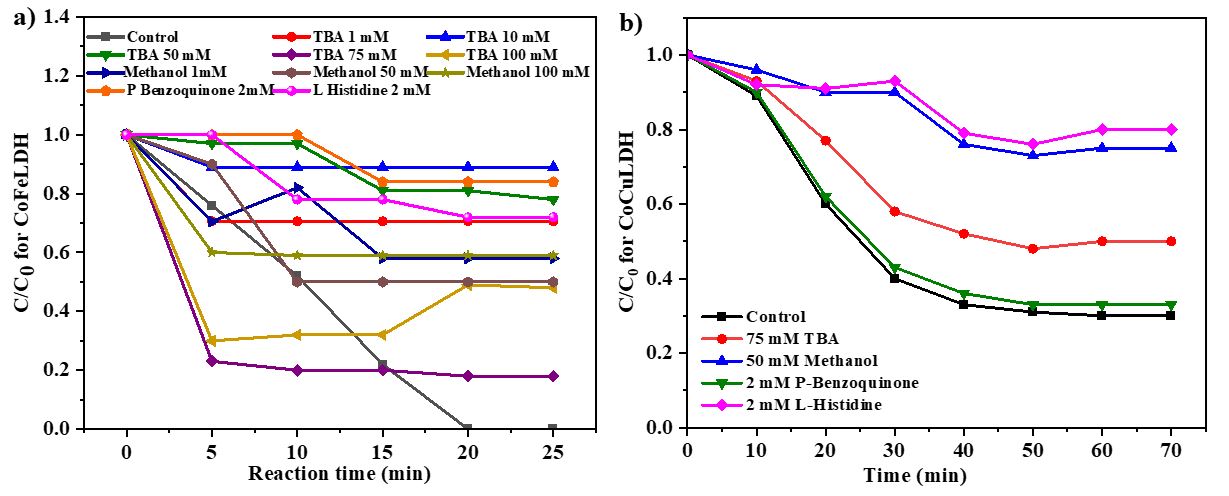
**

Fig. S1: Factorial effects of TBA, methanol, P-Benzoquinone and L-Histidine for the catalytic degradation of ranitidine a) by CoFeLDH catalyst and b) by CoCuLDH catalysts. Experimental condition: LDH 20 mg/L, PMS 20 mg/L, ranitidine concentration 5 mg/L, pH 5 (unadjusted). (The standard deviation among triplicate samples remained less than 5%).

Fig.S2: Ranitidine sample positive gradient mass spectrum at 1.65 min.

Fig. S3: Ranitidine sample positive gradient mass spectrum at 1.482 min.

Fig. S4: Ranitidine sample positive gradient mass spectrum at 1.37 min.
